# *Punica granatum* Peel and Leaf Extracts as Promising Strategies for HSV-1 Treatment

**DOI:** 10.3390/v14122639

**Published:** 2022-11-26

**Authors:** Asma EL-Aguel, Rosamaria Pennisi, Antonella Smeriglio, Imen Kallel, Maria Pia Tamburello, Manuela D’Arrigo, Davide Barreca, Ahmed Gargouri, Domenico Trombetta, Giuseppina Mandalari, Maria Teresa Sciortino

**Affiliations:** 1 Research Laboratory Toxicology-Environmental Microbiology and Health (LR17ES06), Faculty of Sciences of Sfax, P.O. Box 1171, Sfax 3000, Tunisia; 2 Department of Chemical, Biological, Pharmaceutical and Environmental Science, University of Messina, Viale Ferdinando Stagno d’Alcontres 31, 98166 Messina, Italy

**Keywords:** *Punica granatum* L., LC-PDA-ESI-MS analysis, antioxidant activity, antimicrobial activity, antiviral activity, herpes simplex virus-1

## Abstract

*Punica granatum* is a rich source of bioactive compounds which exhibit various biological effects. In this study, pomegranate peel and leaf ethanolic crude extracts (PPE and PLE, respectively) were phytochemically characterized and screened for antioxidant, antimicrobial and antiviral activity. LC-PDA-ESI-MS analysis led to the identification of different compounds, including ellagitannins, flavonoids and phenolic acids. The low IC_50_ values, obtained by DPPH and FRAP assays, showed a noticeable antioxidant effect of PPE and PLE comparable to the reference standards. Both crude extracts and their main compounds (gallic acid, ellagic acid and punicalagin) were not toxic on Vero cells and exhibited a remarkable inhibitory effect on herpes simplex type 1 (HSV-1) viral plaques formation. Specifically, PPE inhibited HSV-1 adsorption to the cell surface more than PLE. Indeed, the viral DNA accumulation, the transcription of viral genes and the expression of viral proteins were significantly affected by PPE treatment. Amongst the compounds, punicalagin, which is abundant in PPE crude extract, inhibited HSV-1 replication, reducing viral DNA and transcripts accumulation, as well as proteins of all three phases of the viral replication cascade. In contrast, no antibacterial activity was detected. In conclusion, our findings indicate that *Punica granatum* peel and leaf extracts, especially punicalagin, could be a promising therapeutic candidate against HSV-1.

## 1. Introduction

*Punica granatum* L. (pomegranate), which belongs to the *Punicaceae* family, is widely distributed in tropical regions. In Tunisia, pomegranate has been traditionally cultivated since ancient times and has an important ecological and socio-economic role. The plant is well adapted to arid and semi-arid environments. Its cultivation is spread throughout the country with Gabes, Kairouan, and Testour as the main producing areas [1,2]. As an ancient plant, it has been used worldwide in traditional medicine for the treatment of atherosclerosis, diabetes, hypertension, hyperlipidemia, several types of cancer and oral diseases. Furthermore, for hundreds of years, it has been well known to relieve hepatic failure and as a treatment for gastrointestinal worms and diarrhea. It is becoming increasingly popular due to its beneficial physiological activities, particularly antioxidant, antimicrobial, anti-inflammatory, antidiabetic and chemo-preventive properties [3]. Pomegranate is a rich source of different nutrient and non-nutrient compounds, such as sugars, organic acids, alkaloids, phenolic acids, tannins, flavonoids, anthocyanins, fatty acids and vitamins, which are well distributed in the various parts of the fruit and whole plant (peel, bark, leaves, and flowers) [4,5].

Several studies have experimentally demonstrated the health effects resulting from the traditional use of this plant, including strong antibacterial, antiviral, antioxidant, and anti-inflammatory activity [6,7,8,9,10]. It has been demonstrated that *Punica granatum* extracts have broad-spectrum antibacterial activity against Gram-positive and Gram-negative bacteria, as well as against many fungi [11,12], also in comparison with several synthetic drugs, with low potency, toxicities, and many adverse effects [13]. The antiviral potential is mainly due to the richness of phenolic compounds. Amongst these, punicalagin has been reported to exert a wide range of biological activities including antiviral effects against human immunodeficiency virus (HIV) [14], influenza virus [15] and herpes simplex virus (HSV) [16]. Several studies showed the strong antioxidant capacity of pomegranate and its anthocyanins, which showed higher antioxidant activity than other natural antioxidants such as vitamin E (α-tocopherol), vitamin C and vitamin A [17], paving the way to its potential use as a food additive with significant health value. Finally, an interesting inhibition of lipoxygenase (LOX) and cyclooxygenase (COX) enzymes, well known to be involved in the inflammatory cascade, was recently observed for *Punica granatum* peel extract [18]. These interesting biological activities are due to the richness of secondary metabolites, particularly phenolic compounds, which represent 50% of the pomegranate peel. Amongst these, the most representatives are ellagic acid, punicalagin and gallic acid [19]. Thus, in this work, we first chemically characterized the pomegranate ethanolic extracts by LC-PDA-ESI-MS analysis and then investigated their antioxidant, antimicrobial and antiviral activity through *in vitro* studies. The study was focused on herpes simplex virus 1 (HSV-1), which is frequently involved in human pathologies and responsible for cutaneous, mucosal, and ocular infections affecting mucous membranes of the mouth, nose, or eyes. In addition, it is the most common cause of viral encephalitis in humans worldwide and can manipulate the host immune response to improve viral replication [20,21,22]. The HSV-1 virion consists of the envelope, the tegument and the nucleocapsid. Viral surface glycoproteins mediate the virus’s attachment and penetration into host cells and elicit host immune responses. The viral genome is surrounded from the inside out by nucleocapsids and viral tegument proteins. The entry of HSV-1 into the host cells involves the interaction between at least four glycoproteins (gB, gC, gD, gH and gL) embedded in the envelope with specific cell surface receptors. After its entry, several tegument proteins remain associated with the nucleocapsid and promote the capsid trafficking to the nuclear compartment. Once in the nucleus, the linear genome is converted into a covalently closed “endless” circular form, and both viral genome replication and viral transcription begin. The HSV-1 genome replication is a cascade effect involving the regulated expression of three classes of viral genes: immediate-early (IE), early (E), and late (L) genes. The IE genes, which are expressed following virus internalization, regulate the expression of E and L viral genes. To date, acyclovir (ACV) and its derivatives are the first-line drugs for herpetic infections and target the viral DNA polymerase. However, long-term administration can lead to a substantial level of drug resistance [23]. In the present study, we provide new evidence about the inhibitory activity of PPE against HSV-1 infection and report the underlying mechanism of this activity.

## 2. Materials and Methods

### 2.1. Chemicals

Gallic acid, quercetin, Folin-Ciocalteu reagent, sodium carbonate, aluminum chloride and sodium acetate were purchased from Sigma Aldrich (St. Louis, MO, USA). LC-MS grade acetonitrile, water and formic acid were purchased from Merck (Darmstadt, Germany). High-purity (≥98%) HPLC reference standards (gallic acid, catechin, α-punicalagin, ellagic acid, cyanidin 3-O-glucoside, quercetin, coumaric acid, caffeic acid, ferulic acid, pelargonidin, vanillic acid, kaempferol-3-O-glucoside) were purchased from Extrasynthese (Genay, France).

### 2.2. Plant Material and Extract Preparation

Peels (PP) and leaves (PL) of *P. granatum* of the Gabsi variant were sampled in the south east of Tunisia (33°53′ North, 10°07′ East), and the botanical identification of the plant was confirmed according to *Flore succinte et illustrée des zônes arides et sahariennes de Tunisie* [24,25]. The extract was dried at room temperature for one week until constant mass. The PP/PL powder was extracted by simple maceration with ethanol (500 mL) for 24 h. Then, the mixture was filtered and concentrated under a vacuum at 40 °C by a rotary evaporator. The two ethanolic extracts (PPE and PLE) were stored in refrigerated sterile bottles until further use.

### 2.3. Total Phenols

The total phenols content was determined according to Smeriglio et al. [26]. Briefly, 50 µL of PPE, PLE (0.5–4.0 mg/mL), and gallic acid as the reference standard (0.075–0.60 µg/mL) were added to 500 µL of Folin–Ciocalteu reagent and brought to 1 mL with deionized water. After 3 min, 10% sodium carbonate (500 µL) was added. Samples were left in the dark at room temperature (RT) for 1 h with mixing every 10 min. The absorbance was recorded at 785 nm by an UV–Vis spectrophotometer (Shimadzu UV-1601, Kyoto, Japan). Results were expressed as mg of gallic acid equivalents (GAE)/100 g of dry extract (DE).

### 2.4. Flavonoid Determination

The flavonoid content was evaluated according to Smeriglio et al. [26]. Briefly, 200 µL of PLE, PPE (0.5–4.0 mg/mL), and quercetin as the reference standard (0.125–1.0 mg/mL) were added to 2 mg/mL AlCl_3_ (1:1, *v*/*v*) and brought to 1.6 mL with 50 mg/mL sodium acetate. After 2.5 h, the absorbance was recorded at 440 nm by an UV–Vis spectrophotometer (Shimadzu UV-1601, Kyoto, Japan). The results were expressed as mg of quercetin equivalents (QE)/100 g DE.

### 2.5. Determination of Polyphenolic Profile by LC-PDA-ESI-MS Analysis

The phytochemical analysis of PLE and PPE was carried out using an Agilent high-performance liquid chromatography system (HPLC 1100 series) equipped with an UV–Vis photodiode array (PDA-G1315) detector and an ion trap mass spectrometer detector (IT-6320). The electrospray ionization (ESI) source was used in full scan mode, monitoring the precursor ions between *m*/*z* 50 and *m*/*z* 1500 in negative polarity using the following parameters: capillary voltage, 3.5 kV; drying gas temperature, 350 °C; nitrogen flow, 10 L/min; and nitrogen pressure, 50 psi. Data processing was carried out by Agilent 6300Series Ion Trap LC/MS system software (version 6.2). The chromatographic separation was achieved by a Luna Omega PS C18 column (150 mm × 2.1 mm, 5 µm; Phenomenex, CA, USA) using solvent A (0.1% formic acid) and solvent B (acetonitrile) as the mobile phase. The elution program was as follows: 0–3 min, 0% B; 3–9 min, 3% B; 9–24 min, 12% B; 24–30 min, 20% B; 30–33 min, 20% B; 33–43 min, 30% B; 43–63 min, 50% B; 63–66 min, 50% B; 66–76 min, 60% B; 76–81 min, 60% B; 81–86 min, 0% B and equilibrated 4 min for a total run time of 90 min. The flow rate was 0.4 mL/min, and the column temperature and the injection volume were 25 °C and 5 µL, respectively. UV–Vis spectra were recorded in the range of 190–700 nm, and chromatograms were acquired at 254, 280, 340, 370, and 520 nm. The acquisition wavelength chosen to show and compare the phytochemical profile of both extracts, at which all the identified peaks were visible, was 254 nm. The peaks were identified by comparing the retention time, mass, and UV–Vis spectra with literature data and, when available, with reference standards (gallic acid, catechin, α-punicalagin, ellagic acid, cyanidin 3-*O*-glucoside, quercetin, coumaric acid, caffeic acid, ferulic acid, pelargonidin, vanillic acid, and kaempferol-3-*O*-glucoside).

### 2.6. Antioxidants Assay

#### 2.6.1. DPPH Radical Scavenging Assay

The DPPH free radical scavenging activity of the extracts was evaluated according to Kirby and Schmidt [27]. Briefly, reaction mixtures containing 0.5 mL of sample (0.125–1.0 mg/mL) were mixed with 1 mL of a DPPH ethanol solution (0.04 mg/mL). The samples were kept in the dark for 20 min at room temperature and the absorbance was measured using a spectrophotometer against a blank at 517 nm. The inhibition percentage was calculated as:

(1) I(%)=[(A0−A1)/A0)]×100

where A0 is the absorbance of the blank and A1 is the absorbance of the sample. BHT was used as a positive control [28].

#### 2.6.2. Ferric Reducing Antioxidant Power Assay (FRAP Assay)

The reducing power was determined according to the Oyaizu method [29] with slight modifications. Briefly, 250 μL of the sample (0.125–1.0 mg/mL) was mixed with a phosphate buffer (500 μL, 0.2 M, pH 6.6) and potassium ferricyanide (500 μL, 1%). The mixtures were then incubated in a water bath at 50 °C for 20 min. Then, 500 μL of trichloroacetic acid (10%) was added to each sample, and the mixture was centrifuged at 1000 rpm for 10 min. After that, 750 μL of the upper layer was mixed with 750 μL of distilled water and 50 μL of ferric chloride (0.1%) was added. The absorbance was recorded at 700 nm against a blank consisting of all reagents without the test sample and by using ascorbic acid as a reference compound [30].

### 2.7. Cells Culture and Virus

Vero cell lines (American Type Culture Collection) were propagated in minimal essential medium (EMEM) and supplemented with 6% fetal bovine serum (FBS) (Lonza, Belgium) at 37 °C under 5% CO_2_. The prototype HSV-1 (F) strain was kindly provided by Dr. Bernard Roizman (University of Chicago, Chicago, IL, USA), and the virus stock was produced and titered in Vero cells.

### 2.8. Cell Proliferation Assay

The Vero cells were grown in wells of 96-well plates and treated with different concentrations of PPE and PLE (25, 50 and 100 µg/mL) for 24, 48 and 72 h and punicalagin, ellagic acid, and gallic acid (25 µg/mL, 20µg/mL, 10µg/mL, 5 µg/mL and 2.5 µg/mL) for 72 h. Similarly, the cells were treated with 1, 10, 20, 50, 100, 200 and 500 µM of Acyclovir, and the cellular viability was tested 24 h post-treatment. The cell viability following treatment was measured using the ViaLightTM plus cell proliferation and cytotoxicity bioassay kit according to the manufacturer’s instructions (Lonza Group Ltd., Basel, Switzerland). The luminescence values were measured by GloMax^®^ Multi Microplate Luminometer (Promega Corporation, 2800 Woods Hollow Road, Madison, WI, USA) and converted into the cell proliferation index (%) as previously reported [31].

### 2.9. Plaque Reduction Assay

The Vero cells were seeded on 24-well plates and infected with the virus inoculum for 1 h at 37 °C with gentle shaking. The virus stock was serially diluted to yield 60 plaques/1000 µL. Both the virus and cells were pretreated for 1 h at 37 °C with 25, 50 and 100 µg/mL of PPE and PLE, separately. Similarly, the Vero cells and viral dilution were incubated with punicalagin (20 µg/mL, 10 µg/mL, 5 µg/mL and 2.5 µg/mL), ellagic acid and gallic acid (10 µg/mL, 5 µg/mL and 2.5 µg/mL) for 1h. The infection was then performed at 1 MOI with gentle shaking. Thus, the pretreated virus inoculum was added to pretreated Vero cells to promote viral adsorption. Thus, 1 h later, the monolayer was covered with a medium containing 0.8% methylcellulose in the presence of PPE and PLE crude extracts and compounds. DMSO was included as a control and used at the highest concentration (50 µM). After three days, the cells were fixed, stained with crystal violet, and visualized with an inverted microscope (Leica DMIL, Nuloch, Germany) for plaque detection. The data were analyzed as triplicates ± standard deviation (SD) for each dilution.

### 2.10. The Binding Assay

The binding assay was performed under two conditions to investigate whether the extracts affected the first step of viral infection acting on the virus or the cell surface. Thus, the pretreatment of the virus was performed before infection and separately the cells were treated before with the extracts and then infected with the virus. In both cases, pretreatment was performed with 50 µg/mL and 100 µg/mL of PPE and PLE for 1 h. Whereupon, the Vero cells (4 × 10^5^ cells/well) were infected with pretreated HSV-1 virus at a multiplicity of infection (MOI) of 1 PFU/cell, and pretreated Vero cells (4 × 10^5^ cells/well) were infected with the untreated HSV-1 virus. The viral infection was performed at 4 °C for 1 h to allow the binding of the virus, but not the penetration into the cells. The virus inoculum was then removed and the monolayers were washed three times with medium and replaced with medium containing 0.8% methylcellulose or growth medium according to experiments. For viral DNA, viral genes quantification and Western blot analysis, samples were collected at 24 h post-infection and processed. DMSO was used as a control at 50 µg/mL.

### 2.11. Protein Extraction and Immunoblot Analysis

Immunoblot analysis was carried out to evaluate the accumulation of viral proteins. Samples were lysed in SDS sample buffer 1X (62.5 mM Tris-HCl (Tris(hydroxymethyl)aminomethane hydrochloride) pH 6.8; 50 mM DTT (dithiothreitol); 10% glycerol; 2% SDS (sodium dodecyl sulfate); 0.01% Bromophenol Blue; EDTA-free Protease Inhibitor Cocktail 1X (Roche)), sonicated, boiled for 5 min and analyzed for protein determination using a QubitTM Protein Assay Kit (InvitrogenTM). An equal amount of protein extract was subjected to SDS-gel electrophoresis (SDS-PAGE) and transferred to membranes (Bio-Rad Life Science Research, Hercules, CA, USA). The membranes were incubated overnight at 4 °C with the appropriate primary antibody and then probed with secondary antibodies at room temperature for 1 h. Specific bands were visualized using Immobilon Classico Western HRP substrate (Merk, Millipore). Anti-GAPDH (sc-32233), anti-HSV-1 UL42 (sc-53333), and anti-ICP0 (sc-56985) were purchased from Santa Cruz Biotechnology (Santa Cruz, CA, USA). Monoclonal anti-US11 was provided by Professor Bernard Roizman. Secondary HRP-conjugated goat anti-mouse IgG was purchased from Millipore. Quantitative densitometry analysis of immunoblot band intensities was performed using ImageJ software. The intensity of the target protein was divided by the intensity of the GAPDH and graphically represented by GraphPad Prism 6 software (GraphPad Software, San Diego, CA, USA). Statistical analysis was performed by ANOVA followed by Bonferroni’s Multiple Comparison Test.

### 2.12. Viral DNA Extraction and Real Time-PCR

Viral DNA was extracted with phenol/chloroform solution and precipitated from the organic phase. The DNA pellet was washed twice in a solution containing 0.1 M trisodium citrate in 10% ethanol and then dissolved in 8 mM NaOH. The concentration of DNA was determined by fluorometer analysis with the Qubit double-stranded DNA (dsDNA) HS (High Sensitivity) Assay Kit according to the manufacturer’s instructions. The amplification of viral DNA was carried out by TaqMan™ Universal Master Mix II (Applied Biosystems™, Foster City, CA, USA) in a 50 µL reaction mixture containing the following: TaqMan Universal Master Mix II, DNA (100 ng); HSV-1 forward (10 µM) and HSV-1 reverse (10 µM) primers (Fw 5′-catcaccgacccggagagggac; Rev 5′-gggccaggcgcttgttggtgta); and the TaqMan probe (5 µM) (5′-6FAM-ccgccgaactgagcagacacccgcgc-TAMRA, where 6FAM is 6-carboxyfluorescein and TAMRA is 6-carboxytetramethylrhodamine). The amplification was carried out on Applied Biosystems 7300 Real-Time PCR System under the following conditions: 10 min at 95 °C, 60 s at 95 °C for 40 cycles, 30 s at 60 °C, and 30 s at 72 °C. Each amplification run contained one negative control. The primers were designed on the large catalytic subunit of HSV-1 DNA polymerase holoenzyme (*ul30* gene). The relative quantitation of HSV-1 DNA was generated by the comparative Ct method using GAPDH as a housekeeping gene. One-way analysis of variance (ANOVA) and the GraphPad Prism 6 software (GraphPad Software, San Diego, CA, USA) was used to perform statistical analysis and graphical representations, respectively.

### 2.13. RNA Extraction and Real Time-PCR

Total RNA was extracted using TRIzol^®^ (Thermo Fisher Scientific, Waltham, MA, USA) according to the manufacturer’s instructions and DNase-treated before cDNA transcription as follows: 1 μg of RNA was incubated at 37 °C for 2 h with 5 μL 10X DNase I Buffer, 2 μL Recombinant RNase-free DNase I (10U) (2270A TaKaRa, Dalian, China) and RNase inhibitor (20U) (N251A Promega).

The samples were treated with 2.5 μL EDTA 0.5 M for 2 min at 80 °C and stored in the presence of 10 μL sodium acetate 3 M and 250 μL cold ethanol at −80 °C. Next, samples were centrifuged at 12,000 rpm for 10 min at 4 °C. RNA pellets were then washed with 70% cold ethanol, centrifuged at 12,000 rpm for 5 min at 4 °C, air-dried at RT to remove ethanol and dissolved in a suitable amount of DEPC-treated water. Total RNA (1 μg) was reverse transcribed using ReverTra Ace^®^ qPCR RT Master Mix (FSQ-201 Toyobo). The retrotranscription reaction was carried out in a BiometraTPersonal PCR thermocycler under the following conditions: 37 °C for 15 min, followed by 50 °C for 5 min and 98 °C for 5 min. The cDNAs were used for quantitative Real-Time PCR carried out using Applied Biosystems 7300 Real-Time PCR System. The analytic primers for RT-PCR are the following: ICP0 Fw-5′-tctgcatcccgtgcatgaaaac-3′ and Rev-5′-ctgattgcccgtccagataaag-3′; UL42 Fw-5′-ctccctcctgagcgtgtttc-3′ and Rev-5′-cacaaagctcgtcagttcgc -3′; and Us11 Fw-5′-ggcttcagatggcttcgag -3′ and Rev-5′-gggcgacccagatgtttac-3′. The thermal profile consisted of a 10 min incubation at 95 °C followed by 30 cycles of 15 s denaturation at 95 °C, 35 s annealing at 60 °C and 45 s elongation at 72 °C.

### 2.14. Antimicrobial Assay

#### 2.14.1. Microbial Strains and Culture Conditions

A range of strains obtained from the University of Messina’s in-house culture collection (Messina, Italy) was used for the susceptibility studies: *Staphylococcus aureus* ATCC 6538, *Escherichia coli* ATCC 10231, *Pseudomonas aeruginosa* ATCC 9027and *Candida albicans* ATCC 10231. All the bacterial strains were grown in Mueller–Hinton Broth (MHB, Oxoid, CM0405) at 37 °C (18–20 h), and *C. albicans* was cultured in RPMI 1640 at 30 °C (24–48 h).

#### 2.14.2. Susceptibility Assays

The minimum inhibitory concentration (MIC) was determined by the broth microdilution method according to the Clinical and Laboratory Standards Institute (CLSI) for bacteria [32] and yeasts [33]. A positive control of an antibiotic or an antifungal was included in each assay. The MIC value was defined as the lowest concentration able to inhibit bacterial or fungal growth.

### 2.15. Statistical Analysis

Three independent experiments were carried out in triplicate (n = 3) for each assay and the results represent the average ± SD. The statistical analysis was performed with GraphPad Prism 8 software (Graph-Pad Software, San Diego, CA, USA) using one-way variance analysis (ANOVA). The significance of the *p*-value is indicated with asterisks (*, **, ***, ****), denoting the *p*-value significance levels less than 0.05, 0.01, 0.001, and 0.0001, respectively. The half-maximal cytotoxic concentration (CC_50_) and the half-maximal effective concentration (EC_50_) values were calculated using non-linear regression analysis.

## 3. Results

### 3.1. Phytochemical Analysis

**Figure 1:**
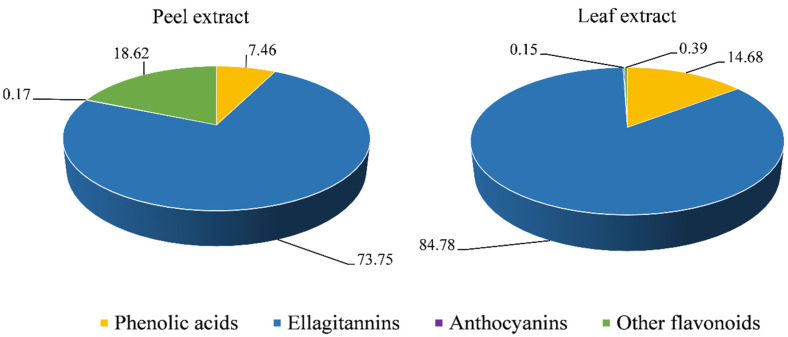
Main classes of polyphenols (%) identified and quantified in pomegranate peel and leaf extract by LC-PDA-ESI-MS analysis.

The *in vitro* phytochemical screening highlighted a very high content in both PPE and PLE of total phenols (282.88 ± 12.55 mg GAE/g DE and 352.56 ± 18.32 mg GAE/g DE, respectively) and flavonoids (288.22 ± 15.44 mg QE/g DE and 177.55 ± 9.58 mg GAE/g DE, respectively), with PPE showing the best content of flavonoids and PLE showing the highest total phenols content. These preliminary results have been corroborated by the LC-PDA-ESI-MS analysis, as shown in Figure 1.

Results of the tentative identification and quantification of polyphenols detected in PPE and PLE are reported in Table 1.

Ellagitannins represent the main polyphenolic group in both extracts, with PLE showing the highest content (84.78%). In PLE, this was followed by phenolic acids (14.68%) including gallic, caffeic and coumaric acid derivatives, flavonoids (0.39%) including phlorizin, and anthocyanins (0.15%) such as cyanidin and delphinidin derivatives as well as pelargonidin. In contrast, PPE was found to be richer in flavonoids (18.62%) such as quercetin, catechin and kaempferol derivatives, the most abundant polyphenols class after ellagitannins (73.75%). These were followed by phenolic acids (7.46%), mainly gallic acid derivatives, and anthocyanins (0.17%). α-Punicalagin and β-punicalagin were the most abundant ellagitannins detected in PPE (21,345.66 and 22,322.88 mg/kg, respectively) followed by ellagic acid hexoside (18,765.33 mg/kg), ellagic acid (13245.66 mg/kg) and ellagic acid pentoside (11,234.66 mg/kg). Conversely, the most abundant compounds detected in PLE were the ellagitannins ellagic acid hexoside (20123.67 mg/kg), ellagic acid dihexoside (3245.66 mg/kg), ellagic acid deoxyhexoseide (2467.88 mg/kg) and the phenolic acids gallic acid (2134.55 mg/kg) and galloyl-HHDP-hexoside (1021.33 mg/kg).

### 3.2. Antioxidant Activity

**Figure 2:**
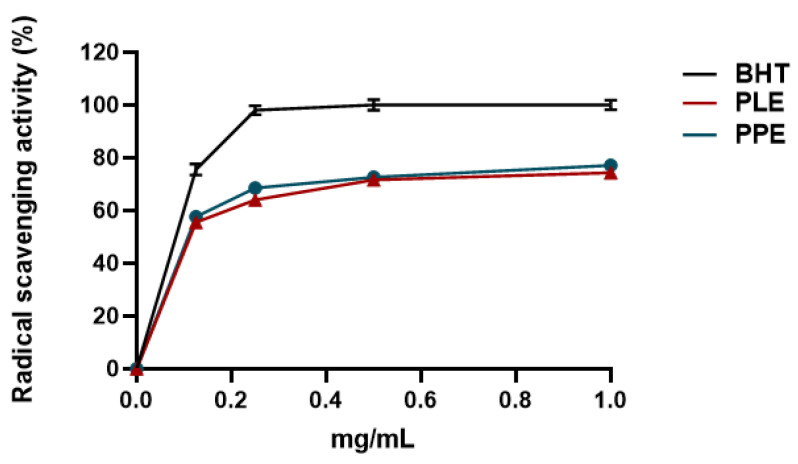
Percentage of radical scavenging capacity for PPE and PLE. Results were expressed as mean radical scavenging activity percentage (%) ± standard deviations (n = 3). BHT was used as the reference standard.

**Figure 3:**
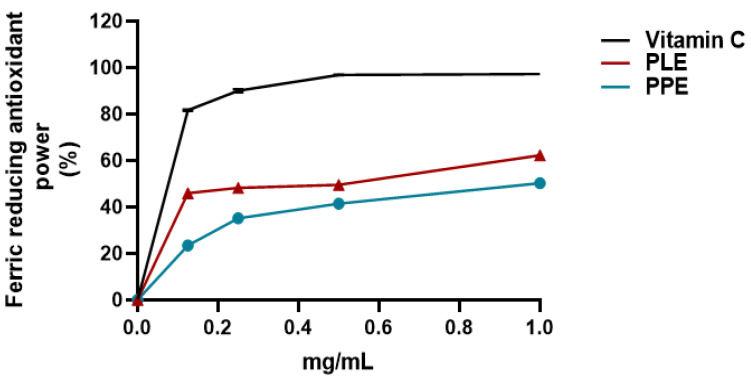
Ferric reducing antioxidant power (FRAP) assay of PPE and PLE. Results were expressed as mean radical scavenging activity percentage (%) ± standard deviations (n = 3). Ascorbic acid was used as the reference standard.

The antioxidant capacity of PPE and PLE was determined according to the DPPH free radical scavenging assay and FRAP assay. Two standard antioxidants (i.e., BHT and ascorbic acid) were used as positive controls for the DPPH free radical scavenging assay and FRAP assay, respectively. The scavenging effect of both extracts was calculated as reported in Section 2.6.1 and Section 2.6.2, and the results were expressed as a percentage of radical scavenging activity and ferric-reducing antioxidant power, respectively. Figure 2 shows that PPE and PLE possessed a striking antiradical activity (77% and 74%, respectively) at 1 mg/mL. The concentration of the PPE and PLE that can scavenge 50% of DPPH free radical (IC_50_) was also determined. The lower IC_50_ value implies a higher antioxidant activity. PPE and PLE have similar IC_50_ values (0.108 mg/mL and 0.112 mg/mL) comparable with that of the reference standard BHT (IC_50_ = 0.082 mg/mL), suggesting their antioxidant effectiveness (Table 2). The antioxidant activity of PPE and PLE was further confirmed by the FRAP assay that measures the ability of the sample to reduce Fe^3+^ to Fe^2+^. Various concentrations of both extracts (0.125–1.0 mg/mL) and ascorbic acid were mixed with FRAP reagents according to the procedure reported above. Given that the FRAP assay is a redox-linked colorimetric reaction, the reduction of ferric ion (Fe^3+^) to ferrous form (Fe^2+^) by antioxidants produces an intense blue light revealed as a change in absorption at 593 nm. Like the antioxidant activity of ascorbic acid, the reducing power of both extracts moderately increased with increasing concentration, reaching 1 mg/mL, 62% for PLE and 50% for PPE (Figure 3). The IC_50_ values of PPE and PLE for the FRAP assays were reported in Table 2.

### 3.3. Cytotoxicity of Pomegranate Crude Extracts on Cell Cultures

**Figure 4:**
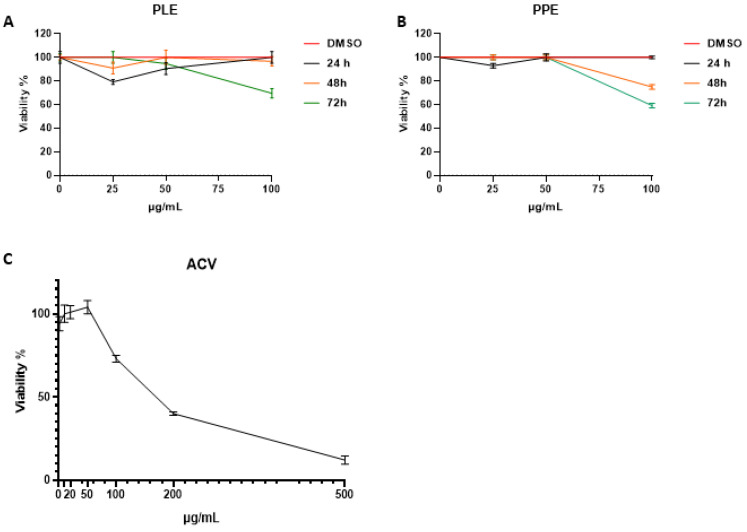
Viability assay in Vero cells treated with *P. granatum* ethanolic extracts (PLE and PPE). (**A**,**B**) Vero cells were treated with different concentrations of pomegranate extracts (25 µg/mL, 50 µg/mL and 100 µg/mL). The cells were collected 24, 48 and 72 h post-treatment. The cell viability was carried out as described in Section 2 and graphically reported as proliferation index (%). (**C**) Cytotoxic effect of acyclovir against Vero cells. The cells were treated with 1, 10, 20, 50, 100, 200 and 500 µM of ACV and the cellular viability was tested 24 h post-treatment. The results are presented as the means of triplicates ± SD. PLE: pomegranate leaf ethanolic extract; PPE: pomegranate peel ethanolic extract; ACV: acyclovir.

To evaluate the cytotoxic effect of PPE and PLE, Vero cells were incubated in the presence of different concentrations of both extracts for 24, 48 and 72 h. The samples were then collected and ATP levels, related to the functional integrity of living cells, were measured. The results are reported as a percentage of cellular viability (Figure 4). Both extracts did not show a marked cytotoxic effect except for the prolonged exposition of 72 h at 100 µg/mL, which moderately affected cellular proliferation. Indeed, PLE displayed cytotoxicity against Vero cells at 72 h with a CC_50_ value of 101 µg/mL and PPE at 48 h and 72 h with CC_50_ of 130 µg/mL. Overall, the results indicate that treatment with pomegranate extracts did not exhibit inhibition of cell growth (Figure 4 A, B). In addition, the cytotoxic effect of acyclovir against Vero cells was tested. The results, shown in Figure 4C, are also consistent with previous findings.

### 3.4. Antiviral Effect of PLE and PPE

**Figure 5:**
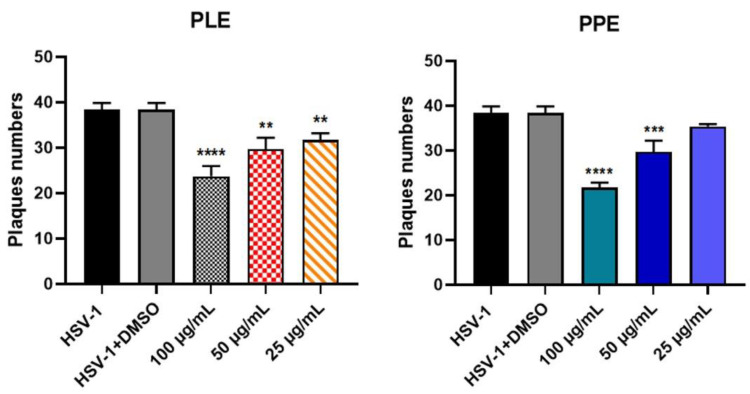
Plaque reduction assay. Results are the mean ± SD of triplicate experiments, and asterisks indicate a significant *p*-value (** *p* < 0.01, *** *p* < 0.001 and **** *p* < 0.0001). PLE, pomegranate leaf ethanolic extract; PPE, pomegranate peel ethanolic extract.

The anti-HSV effects of PLE and PPE were investigated in vitro by a plaque reduction assay. The selectivity index (SI) was determined by the ratio of CC_50_ to EC_50_ (Figure 5, Table 3). PLE and PPE displayed significant antiviral effects against HSV-1 (*p* < 0.001 and *p* < 0.0001).

### 3.5. PLE and PPE Prevent the Binding of HSV-1 on Vero Cells

**Figure 6:**
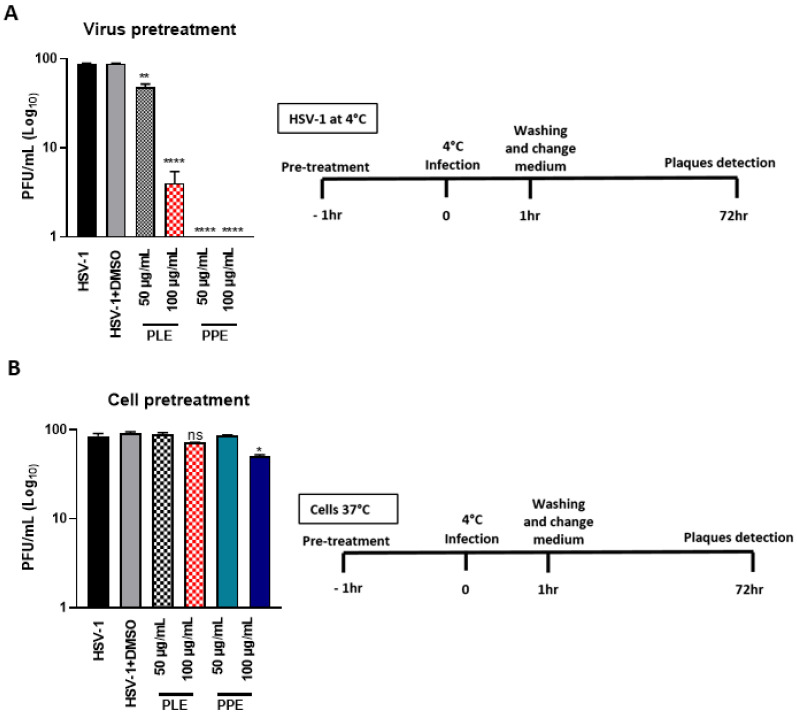
Binding assay following PPE and PLE treatment. Binding experiments were carried out at 4 °C to allow cellular binding but not internalization of the virus and were performed under two conditions: (**A**) pretreatment of the virus before infection (virus pretreatment) and (**B**) pretreatment of cells with the extracts before infection with the virus (cell pretreatment). Vero cells and viral dilution were separately incubated for 1 h at 4 °C with or without PPE and PLE at 100 µg/mL and 50 µg/mL. Then, the infection was performed at a multiplicity of infection (MOI) of 1 with gentle shaking. After 1 h, the virus inoculum was removed, and the monolayers were differentially overlaid with culture medium containing 0.8% methylcellulose for 72 h for the viral plaque reduction assay. Viral plaques were visualized after 3 days using crystal violet staining and visualized with an inverted microscope (Leica DMIL, Nuβloch, Germany) for plaque morphology detection. Graphical representations of binding inhibition assay are shown on the right. * *p* < 0.05, ** *p* < 0.01, **** *p* < 0.0001 vs. HSV-1 + DMSO.

**Figure 7:**
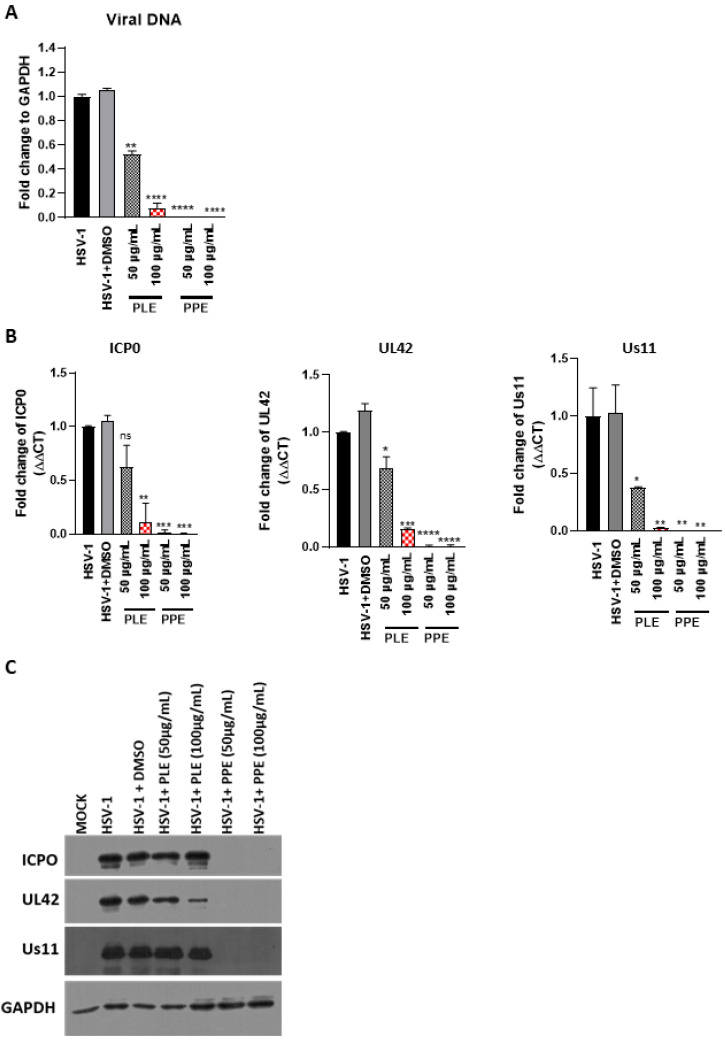
Antiviral activity evaluation of PLE and PPE against HSV-1 downstream of the viral binding. Viral dilution was incubated for 1 h at 4 °C with or without PPE and PLE at 100 µg/mL and 50 µg/mL. Then, the infection was performed on Vero cells at a multiplicity of infection (MOI) of 1 with gentle shaking. After 1 h, the virus inoculum was removed, the monolayer was overlaid with a growth medium for 24 h and processed for real-time PCR and Western blot analysis. (**A**,**B**) Relative quantization of viral DNA and viral transcripts (*ICP0*, *UL42* and *US11*) was performed using real-time quantitative PCR and analyzed by the comparative Ct method (ΔΔCt). Values represent ± SD of the average of three independent experiments normalized against GAPDH. (**C**) Western blot analysis of ICP0, UL42 and Us11 viral proteins. GAPDH was used as a housekeeping gene. The quantitative densitometric analysis for ICP0, UL42 and Us11 band intensities was determined using ImageJ software and expressed as fold change over the appropriate housekeeping gene. * *p* < 0.05, ** *p* < 0.01, *** *p* < 0.001, **** *p* < 0.0001 vs. HSV-1 + DMSO.

The plaque reduction assay showed that PLE and PPE significantly reduced the plaque number. In order to evaluate whether PLE and PPE reduce viral replication by inhibiting the attachment of HSV to cell surfaces, the binding assay was performed under two conditions: (a) pretreatment of the virus before infection (virus pretreatment) and (b) cells treated with the extracts and then infected with the virus (cell pretreatment). The viral infection was performed at 4 °C for 1 h. The effectiveness of both types of extracts was evaluated by the plaque reduction assay. The results clearly show that both extracts were most effective when incubated with HSV-1 before infection on the cell monolayer (Figure 6A vs. Figure 6B) with an EC50 for PPE and PLE of 12.08 µg/mL and 15.79 µg/mL, respectively. Thus, accordingly, to CC50 reported in Table 3, the SI is 8.4 for PPE and 8.2 for PLE. Besides, the binding inhibition between the virus and cells had significant effects on stages of viral infection downstream to viral entry. Indeed, quantitation of viral DNA (Figure 7A), viral transcripts (Figure 7B) and accumulation of viral proteins (Figure 7C) were reported. The results show the accumulation of viral DNA was strongly affected by PPE treatment at both concentrations. On the other hand, PLE reduced viral DNA accumulation only at a high concentration (100 µg/mL) (Figure 7A). PLE and PPE significantly inhibited the expression of HSV-1 immediate-early (IE) *ICP0*, early (E) *UL42* and late (L) *Us11* genes. In particular, PPE treatment strongly affected the transcription of the three representative HSV-1 genes (*ICP0*, *UL42* and *Us11*) more than PLE (Figure 7B). To further determine whether the capability of PLE and PPE to hinder the transcription of viral genes leads to the suppression of HSV-1 protein expression, we evaluated the impact of both extracts on viral proteins ICP0, UL42 and Us11, which are representative products of the viral gene cascade. As expected, Western blot analysis showed that PLE and PPE inhibited the protein expression of HSV-1 (Figure 7C). PPE strongly reduced the ICP0, UL42 and US11 expression, whereas PLE affected the UL42 expression only at higher concentrations. Overall, the results show that PPE, differently from PLE, inhibits the binding of the HSV-1 to the cell surface, allowing the reduction in the viral spread.

### 3.6. Antiviral Activity of PPE and PLE Compounds

**Figure 8:**
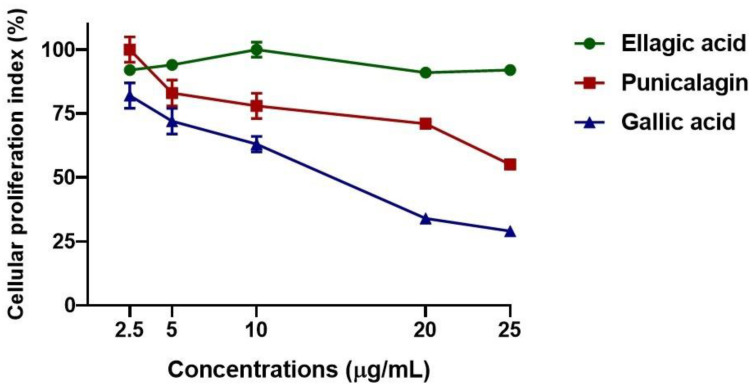
Cytotoxicity assay. Vero cells were treated with different concentrations of punicalagin, ellagic acid, and gallic acid (25 µg/mL, 20 µg/mL, 10 µg/mL, 5 µg/mL and 2.5 µg/mL). The cells were collected 72 h post-treatment, and the cell viability was carried out as described in Section 2. The results are shown graphically as the proliferation index (%). The results presented are the means of triplicates ± SD.

**Figure 9:**
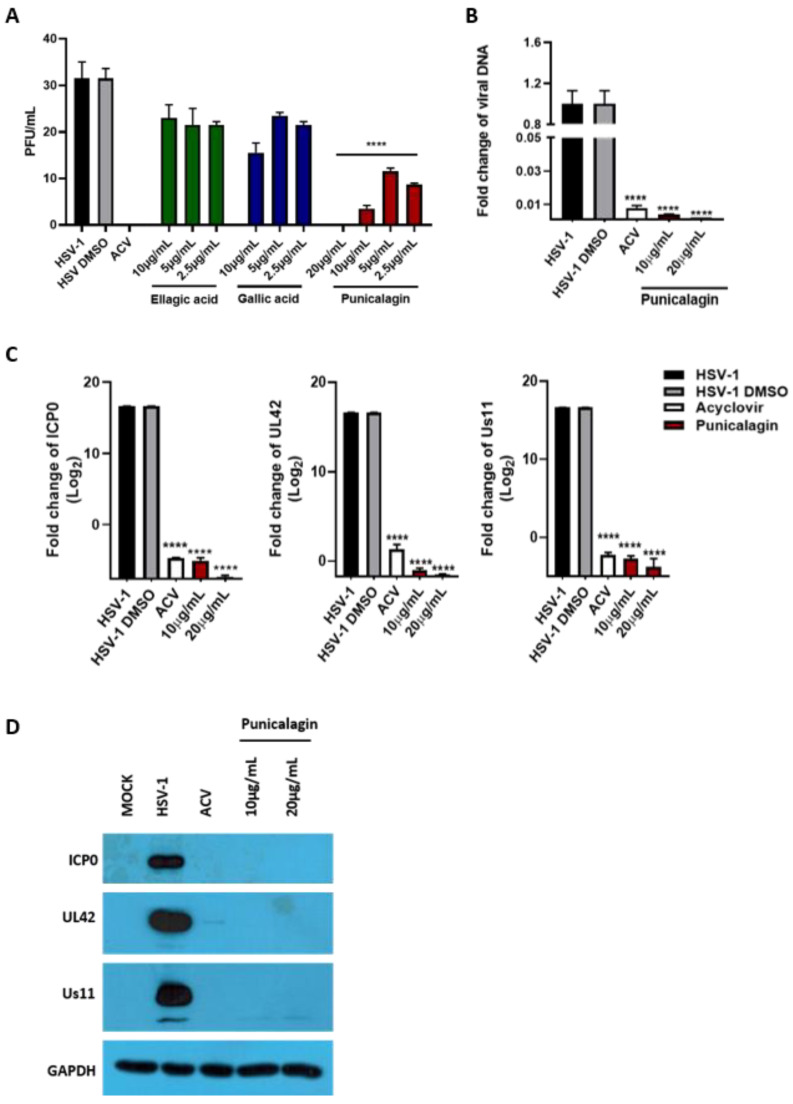
Antiviral activity of pure compounds. (**A**) Vero cells and viral dilutions were incubated with punicalagin (20 µg/mL, 10 µg/mL, 5 µg/mL and 2.5 µg/mL), ellagic acid and gallic acid (10 µg/mL, 5 µg/mL and 2.5 µg/mL) for 1 h. The infection was then performed at 1 MOI with gentle shaking. After 1 h, the virus inoculum was removed and the monolayers were overlaid with a medium containing 0.8% methylcellulose in the presence of the tested compounds. Acyclovir (50 µM) was used as a positive control. The plaques were visualized after 3 days using crystal violet staining. (**B**,**C**) Relative quantification of viral DNA and viral transcripts (ICP0, UL42 and US11) was performed using real-time quantitative PCR and analyzed by the comparative Ct method (ΔΔCt). Values represent ± SD of the average of three independent experiments normalized against GAPDH. (**D**) Western blot analysis of ICP0, UL42 and Us11 viral proteins. GAPDH was used as a housekeeping gene. Data are expressed as a mean (± SD) of at least three experiments. **** *p* < 0.0001 vs. HSV-1 + DMSO.

To provide insights into which of the PPE polyphenols were more relevant than those in PLE for viral inhibition, the three most abundant compounds of PPE and PLE were individually tested. Before exploring the antiviral activity, a viability assay was carried out by treating Vero cells with punicalagin, ellagic acid, and gallic acid at 25 µg/mL 20 µg/mL, 10 µg/mL, 5 µg/mL and 2.5 µg/mL (Figure 8). The results showed a good profile of tolerability to all concentrations of ellagic acid (from 5 µg/mL to 25 µg/mL). In contrast, the punicalagin and gallic acid were toxic at higher concentrations tested. Thus, non-toxic concentrations of punicalagin, ellagic acid, and gallic acid were employed to evaluate the antiviral activity (Figure 9A). The results show that punicalagin, more than ellagic and gallic acid, reduced the viral plaque in a concentration-dependent manner. The half-maximal effective concentration (EC_50_) was calculated from concentration-effect curves by using non-linear regression analysis. The EC_50_ values, CC_50_ and SI are shown in Table 4. Significant reductions in viral DNA (Figure 9B) and transcripts (Figure 9C), as well as proteins of all three phases of the viral replication cascade, were detected following treatment with 20 µg/mL and 10 µg/mL of punicalagin (Figure 9D). These results demonstrate that the antiviral activity of PPE is mainly attributable to the punicalagin content.

### 3.7. Antimicrobial Potential

No activity was shown by PPE and PLE against the tested strains at the concentrations used, which ranged between 1000 and 3.9 mg/mL (results not shown). Results of negative controls confirmed the absence of inhibition by the solvent used (results not shown).

## 4. Discussion

*Punica granatum L*., belonging to the *Punicaceae* family, is one of the most famous and oldest known species worldwide. *P. granatum* is an herb frequently used in Chinese traditional medicine. Its roots, flowers, fruits, peel, seeds, and leaves can be used as medicinal compounds. From a phytochemical point of view, several studies are available in the literature on the polyphenolic composition of pomegranate extracts, although most of these are focused on the edible part (pulp and juice) or the waste products of fruit processing, for example, the peel [34,35,36,37,38,39]. Fewer studies are available on the phytochemical characterization of pomegranate leaf extracts [5,40,41]. Indeed, the total phenols content and flavonoids present in the PLE and PPE significantly exceed (from 2 to 100 times) some data available in the literature [41,42]. Furthermore, almost all examined studies showed a simply qualitative characterization of the extracts under examination by reporting, at most, the relative percentages of the identified compounds. However, from an in-depth analysis, it can be observed that both the extracts examined in this study showed characteristics which are comparable to those present in the current available literature, with ellagitannins representing the most characteristic compounds of the peel, followed by flavonoids, phenolic acids and anthocyanins [34,35,36,37,38,39]. On the other hand, the leaves were characterized not only by a preponderance of ellagitannins, but also by reasonable amounts of phenolic acids and their derivatives as well as by a lower presence of flavonoids [5,40,41]. Only Russo and collaborators [36] carried out a quali-quantitative characterization of the pomegranate extracts by analyzing the juice, pulp and peel of different Italian varieties of *P. granatum*. By examining the data, it can be observed that the quantities of polyphenols found in the peel extract of the present study fall perfectly within the ranges indicated by Russo and collaborators, with values very close to the highest value. Their results, from the quantitative point of view, in relation to the preponderance of some polyphenolic classes over others, are consistent with those of the present study. Indeed, the most abundant class of polyphenols in the peel extract is ellagitannins, with punicalagin and derivatives of ellagic acid being the most representative compounds, followed by the flavonoids catechin, gallocatechin and quercetin derivatives. Govindappa [43] found that silver nanoparticles synthesized from pomegranate pericarp extracts had potent and concentration-dependent antioxidant activity, which is similar to BHT and ascorbic acid with a DPPH inhibitory activity at 1 mg/mL of 81% [41]. Likewise, in the study of Zeghad et al. [44], the antioxidant activity of *P. granatum* peel hydroalcoholic extract from Algeria, established by FRAP and DPPH assays, showed a good antioxidant capacity with an IC_50_ of 2.61 mg/mL and 0.6 mg/mL, respectively. However, in our study, we found much lower IC_50_ values compared with those reported by Zeghad and co-workers [44] (PPE IC_50_ = 0.6 mg/mL and 0.1 mg/mL; PLE IC_50_ = 0.5 mg/mL and 0.11 mg/mL for FRAP and DPPH, respectively). These discrepancies could be due to the different environmental and experimental conditions such as the plant’s development stage, drying temperature, storage and extraction method. In contrast with the findings previously reported [45], which demonstrated that *P. granatum* has significant antimicrobial activities attributed to the presence of various secondary metabolite sources such as steroids, terpenoids, alkaloids, and phenols, no antibacterial activity was observed in the present study for both PPE and PLE. Furthermore, a study by Sun et al. [46] designed to investigate the antimicrobial activity of phenolic compounds from pomegranate peel, revealed that two triterpenes extracted from pomegranate peel (ursolic acid and asiatic acid) showed significant antimicrobial activity against *E. coli*, *S. aureus* and *M. smegmatis*. Additional studies have recently demonstrated that eight different ethanol extracts of pomegranate peels possessed antibacterial activity against *S. aureus* at concentrations ranging from 0.8 to 6.4 mg/mL [47,48,49], which were significantly higher concentrations than those used in the present work. Furthermore, Ismail et al. [50] recorded that the antimicrobial mechanism of pomegranate involves the precipitation of membrane proteins resulting in microbial cell lysis [50]. Nevertheless, as in our study, two other phenolic compounds, corosolic acid and arjunolic acid, did not inhibit the growth of microorganisms at a concentration from 2 to 256 mΜ [46]. According to the polyphenolic profile from the LC-PDA-ESI-MS analysis, the polyphenolic composition of pomegranate extracts changes among the various plant parts such as leaves and peels and could be responsible for different bioactivity [51]. Amongst different biological activities, pomegranate extracts or derivates seemed to exhibit greater potential as antivirals, rather than antibacterials, and can inhibit the replication of viruses belonging to different families with different replicative strategies. Indeed, it is well-known that lyophilized extracts and bioactive compounds isolated from the fruit peel of *P. granatum* exhibit anti-HSV-2 activity and inhibit the influenza virus due to the abundance of punicalagin [52,53]. Pomegranate juice extracts exert antiviral activity against human immunodeficiency virus (HIV), inhibiting the binding of the HIV-1 envelope glycoprotein gp120 to the CD4 cellular receptor [54]. Recently, it has been reported that pomegranate peel extract and its major polyphenols, such as punicalin and punicalagin, attenuate the binding between the SARS-CoV-2 S-glycoprotein and ACE2 receptor [55]. A similar antiviral effect of pomegranate ethanol extract and punicalagin was reported against Mayaro virus (MAYV), an alphavirus belonging to the Togaviridae family [56]. Furthermore, Acquadro et al. [57] demonstrated the anti-Zika activity of pomegranate leaf extract and ellagic acid. This suggests that *P. granatum* extracts possess multiple targets of action. In the context of herpetic infections, aqueous extract from pomegranate leaves exhibited antiviral potency against the Human Herpes Virus-3 (HHV-3) and was highly effective in preventing the HHV-3-induced cytopathic effect in HEp-2 cells [58]. To date, the main challenge for many research groups is discovering novel antagonists/inhibitors of viral replication and expanding the list of candidate antivirals for drug-resistant infections. According to our knowledge, this is the first report that describes the mechanism by which the pomegranate extracts inhibit HSV-1 replication. Our results demonstrate that both PPE and PLE exhibit anti-HSV activity, but mainly PPE inhibits the binding between HSV-1 and the cell surface through virus inactivation. Thus, the inhibition of binding between virus and cells had significant effects on the stages of viral infection downstream to viral entry, as demonstrated by the reductions in viral DNA, viral transcripts and viral protein accumulation. Interestingly, analyzing the polyphenol profile, we found a significant difference in the distribution of bioactive compounds, which may explain the different behavior of PPE and PLE in the inhibition of the binding of HSV to the cell’s surface. Among the main polyphenolic compounds, punicalagin is the most abundant polyphenol and is known for a wide range of biological activities, including antioxidant, anti-atherosclerotic and anti-proliferative activity against tumor cells as well as anti-inflammatory pathways and antiviral activity [59,60]. Clearly, PLE exerts its antiviral activity using an alternative mechanism that probably involves the modulation of cellular processes. For this reason, further studies are needed to better define the mechanisms by which PLE extracts inhibit HSV-1 replication. Collectively, our findings confirm that pomegranate peel extracts, and especially punicalagin, could be of interest as a promising therapeutic candidate against HSV-1 infection.

## 5. Conclusions

In conclusion, our study showed that extracts from pomegranate inhibit HSV-1 replication and have antioxidant potency but no antibacterial effect. Amongst the main polyphenolic compounds, punicalagin appears to be the main component showing effective antiviral activity. The current findings bring new insights and contribute to develop further studies on pomegranate based on its significant antioxidant and antiviral properties.

## Figures and Tables

**Table 1:** Tentative identification and quantification of polyphenols in pomegranate peel and leaf extracts (PPE and PLE, respectively) by LC-PDA-ESI-MS analysis.

No.	Tentative Compound	[M − H]^−^	λ_max_	PPE	PPL
		(*m*/*z*)	(nm)	mg/kg	
1	Gallic acid	169	215, 272	656.55 ^a^	2134.55 ^a^
2	Catechin	289	282	6578.22 ^b^	-
3	Galloyl glucose	331	278	8675.55 ^a^	568.99 ^a^
4	Gallagyl-hexoside (Punicalin)	781	266, 377	678.55 ^c^	-
5	Granatin A	783	272, 366	3088.77 ^c^	-
6	Ellagitannin	643	277	1088.44 ^d^	-
7	Cyanidin 3,5-O-diglucoside	611	515	58.78 ^e^	-
8	Gallocatechin	305	271	4558.77 ^b^	-
9	Phellatin	533	286, 332	4321.99 ^f^	-
10	Granatin B	951	276, 377	876.22 ^c^	143.66 ^c^
11	Phlorizin	435	280	44.88 ^f^	123.67 ^f^
12	Coumaric acid-hexoside	325	314	28.34 ^g^	121.88 ^g^
13	Caffeoyl quinic acid	353	212, 327	-	56.77 ^h^
14	Dicaffeoylquinic acid	515	221, 326	-	79.12 ^h^
15	α-punicalagin	1083	279	21,345.66 ^c^	-
16	Hexahydroxydiphenichexoside	481	267	123.44 ^c^	-
17	Ellagic acid dihexoside	625	288, 320	-	3245.66 ^d^
18	Ellagic acid hexoside	463	255, 362	18,765.33 ^d^	20,123.67 ^d^
19	Feruloyl coniferin	517	227	-	34.88 ^i^
20	Delphinidin-3,5-O-diglucoside	625	516	45.66 ^j^	21.33 ^j^
21	Cyanidin pentoside	417	280, 517	-	23.77 ^e^
22	Cyanidin-3-glucoside	449	274, 532	110.44 ^e^	-
23	β-punicalagin	1083	279	22,322.88 ^c^	-
24	Pelargonidin	271	230, 370	-	2.66 ^k^
25	Delphinidin-3-O-glucoside	465	527	7.88 ^j^	-
26	Vanillic Acid hexoside	329	268,300	654.33 ^l^	-
27	Quercetin 3-O-rhamnoside	447	265, 357	543.22 ^f^	-
28	Ellagic acid deoxyhexoside	447	257, 364	1234.77 ^d^	2467.88 ^d^
29	Hexahydroxydiphenichexoside II (HHDP)	481	268	897.66 ^c^	-
30	bis-HHDP-hexoside (pedunculagin I)	783	260, 382	3012.77 ^c^	534.12 ^c^
31	Castalangin derivative	965	277	-	377.88 ^d^
32	Ellagic acid pentoside	433	256, 362	11,234.66 ^d^	-
33	cis-Dihydrokaempferol hexoside	449	367	2134.76 ^m^	-
34	trans-Dihydrokaempferol hexoside	449	367	1657.22 ^m^	-
35	Galloyl-HHDP-hexoside	633	262, 335	-	1021.33 ^a^
36	Galloyl-HHDP-gluconate	649	267, 377	-	678.99 ^a^
37	Digalloyl-HHDP-glucoside (Punigluconin)	801	270, 377	1023.66 ^c^	145.66 ^c^
38	Ellagic acid	301	255, 367	13,245.66 ^d^	88.99 ^d^
39	Dimethyl ellagic acid glucuronide	505	272, 367	-	-
40	Quercetin hexoside	463	265, 357	3012.67 ^f^	-
41	Kaempferol 3-O-glucoside	447	265, 357	2134.22 ^m^	-

Polyphenols quantification was carried out by the built the following calibration curves: ^a^ gallic acid; ^b^ catechin; ^c^ α-punicalagin; ^d^ ellagic acid; ^e^ cyanidin 3-O-glucoside; ^f^ quercetin; ^g^ coumaric acid; ^h^ caffeic acid; ^i^ ferulic acid; ^j^ delphinidin-3-O-glucoside; ^k^ pelargonidin; ^l^ vanillic acid; ^m^ kaempferol-3-O-glucoside, respectively; -, not detected.

**Table 2:** Half-maximal inhibitory concentration (IC_50_) of PPE and PLE compared with the reference standards BHT and ascorbic acid.

	IC_50_ (mg/mL)			
	BHT	Ascorbic Acid	PLE	PPE
DPPH	0.082	-	0.112	0.108
FRAP	-	0.076	0.504	0.980

IC50: half-maximal inhibitory concentration; BHT: butylhydroxytoluene; PPE: pomegranate peel extract; PLE: pomegranate leaf extract; FRAP: ferric reducing antioxidant power assay; DPPH: free radical scavenging assay.

**Table 3:** Selectivity index (SI), cytotoxicity (CC_50_), and antiviral activity (EC_50_) of PLE and PPE.

Extract	CC_50_ (µg/mL)	EC_50_ (µg/mL)	SI
PLE	130	70.44	1.8
PPE	101	63.76	1.5

CC_50_: half maximal cytotoxic concentration; EC_50_: half maximal effective concentration; SI: selectivity index, the ratio of EC_50_/CC_50_. PPE: pomegranate peel extract; PLE: pomegranate leaf extract.

**Table 4:** Selectivity index (SI), cytotoxicity (CC_50_), and antiviral activity (EC_50_) of punicalagin, ellagic acid and gallic acid.

Extracts	CC_50_ (µg/mL)	EC_50_ (µg/mL)	SI
*Ellagic acid*	184.65	21.69	0.96
*Gallic acid*	19.38	10.93	1.77
*Punicalagin*	29.51	7.97	3.69

CC_50_: half maximal cytotoxic concentration; EC_50_: half maximal effective concentration; SI: selectivity index, the ratio of EC_50_/CC_50_.

## Data Availability

The data presented in this study are available on request from the corresponding authors.

## References

[1] Mansour E., Ben Khaled A., Hadpdapda M., Abid M., Ferchichi A. (2011). Selection of pomegranate (*Punica granatum* L.) in south-eastern Tunisia. Afr. J. Biotechnol..

[2] Jabnoun-Khiareddin H., Ibrahim N., Abdallah R., Mars M., Daami-Remadi M. (2018). Response of Tunisian Pomegranate (*Punica granatum* L.) Cultivars and Several Plant Hosts to Coniellagranati (Saccardo). J. Hortic..

[3] Mirjalili S. (2015). A review on biochemical constituents and medicinal properties of pomegranate (*Punica granatum* L.). J. Med. Plants.

[4] Melgarejo P., Salazar D.M., Artes F. (2000). Organic acids and sugars composition of harvested pomegranate fruits. Eur. Food Res. Technol..

[5] Fellah B., Bannour M., Rocchetti G., Lucini L., Ferchichi A. (2018). Phenolic profiling and antioxidant capacity in flowers, leaves and peels of Tunisian cultivars of *Punica granatum* L.. J. Food Sci. Technol..

[6] Murthy K.N., Jayaprakasha G.K., Singh R.P. (2002). Studies on antioxidant activity of pomegranate (*Punica granatum*) peel extract using in vivo models. J. Agric. Food Chem..

[7] Musarra-Pizzo M., Ginestra G., Smeriglio A., Pennisi R., Sciortino M.T., Mandalari G. (2019). The Antimicrobial and Antiviral Activity of Polyphenols from Almond (*Prunus dulcis* L.) Skin. Nutrients.

[8] Lansky E.P., Newman R.A. (2007). *Punica granatum* (pomegranate) and its potential for prevention and treatment of inflammation and cancer. J. Ethnopharmacol..

[9] Sharifiyan F., Movahedian-Attar A., Nili N., Asgary S. (2016). Study of pomegranate (*Punica granatum* L.) peel extract containing anthocyanins on fatty streak formation in the renal arteries in hypercholesterolemic rabbits. Adv. Biomed. Res..

[10] Musarra-Pizzo M., Pennisi R., Ben-Amor I., Mandalari G., Sciortino M.T. (2021). Antiviral Activity Exerted by Natural Products against Human Viruses. Viruses.

[11] Kamble V.A., Patil S.D. (2008). Spice-derived essential oils: Effective antifungal and possible therapeutic agents. J. HERBS Spices Med. Plants.

[12] Singh B., Singh J.P., Kaur A., Singh N. (2019). Antimicrobial potential of pomegranate peel: A review. Int. J. Food Sci. Technol..

[13] Attia E.A. (2019). Antimicrobial activity and bio-active compounds analysis in ethanolic plant extracts of *Punica grantanum* (pomegranate) using GC-MS. Egypt. J. Exp. Biol..

[14] Nonaka G., Nishioka I., Nishizawa M., Yamagishi T., Kashiwada Y., Dutschman G.E., Bodner A.J., Kilkuskie R.E., Cheng Y.C., Lee K.H. (1990). Anti-AIDS agents, 2: Inhibitory effect of tannins on HIV reverse transcriptase and HIV replication in H9 lymphocyte cells. J. Nat. Prod..

[15] Haidari M., Ali M., Casscells S.W., Madjid M. (2009). Pomegranate (*Punica granatum*) purified polyphenol extract inhibits influenza virus and has a synergistic effect with oseltamivir. Phytomedicine.

[16] Lin L.T., Chen T.Y., Chung C.Y., Noyce R.S., Grindley T.B., McCormick C., Lin T.C., Wang G.H., Lin C.C., Richardson C.D. (2011). Hydrolyzable tannins (chebulagic acid and punicalagin) target viral glycoprotein-glycosaminoglycan interactions to inhibit herpes simplex virus-1 entry and cell-to-cell spread. J. Virol..

[17] Kostka T., Ostberg-Potthoff J.J., Briviba K., Matsugo S., Winterhalter P., Esatbeyoglu T. (2020). Pomegranate (*Punica granatum* L.) Extract and Its Anthocyanin and Copigment Fractions-Free Radical Scavenging Activity and Influence on Cellular Oxidative Stress. Foods.

[18] Sudheesh S., Soumya K., James J. (2018). A novel chalcone derivative from *Punica granatum* peel inhibits LOX/COX enzyme activity. Beni-Suef Univ. J. Basic Appl. Sci..

[19] Spilmont M., Léotoing L., Davicco M.J., Lebecque P., Miot-Noirault E., Pilet P., Rios L., Wittrant Y., Coxam V. (2015). Pomegranate peel extract prevents bone loss in a preclinical model of osteoporosis and stimulates osteoblastic differentiation in vitro. Nutrients.

[20] Whitley R.J. (2006). Herpes simplex encephalitis: Adolescents and adults. Antivir. Res..

[21] Pennisi R., Musarra-Pizzo M., Lei Z., Zhou G.G., Sciortino M.T. (2020). VHS, US3 and UL13 viral tegument proteins are required for Herpes Simplex Virus-Induced modification of protein kinase R. Sci. Rep..

[22] Ghaffari H., Ataei-Pirkooh A., Mirghazanfari S.M., Barati M. (2021). Inhibition of herpes simplex virus type 1 infection by Sambucus ebulus extract in vitro. Med. J. Islam. Repub. Iran.

[23] Piret J., Boivin G. (2016). Antiviral resistance in Herpes simplex virus and Varicella-zoster virus infections: Diagnosis and management. Curr. Opin. Infect. Dis..

[24] Greuter W., Burdet H.M., Long G. (1989). Med-Checklist. A Critical Inventory of Vascular Plants of the Circum Mediterranean Countries.

[25] Chaieb M., Boukhris M. (1998). Flore: Succinte et Illustreé des Zones Arides et Sahariennes de Tunisie.

[26] Smeriglio A., Denaro M., Trombetta D., Ragusa S., Circosta C. (2021). New Insights on *Euphorbia dendroides* L. (Euphorbiaceae): Polyphenol Profile and Biological Properties of Hydroalcoholic Extracts from Aerial Parts. Plants.

[27] Kirby A.J., Schmidt R.J. (1997). Chinese herbs for eczema and of placebo herbs-I. J. Ethnopharmacol..

[28] Elaguel A., Kallel I., Gargouri B., Ben Amor I., Hadrich B., Ben Messaoud E., Gdoura R., Lassoued S., Gargouri A. (2019). Lawsoniainermis essential oil: Extraction optimization by RSM, antioxidant activity, lipid peroxydation and antiproliferative effects. Lipids Health Dis..

[29] Oyaizu M. (1986). Studies on products of browning reaction. Antioxidative activities of products of browning reaction prepared from glucosamine. Jpn. J. Nutr. Diet..

[30] Ben-Amor I., Gargouri B., Attia H., Tlili K., Kallel I., Musarra-Pizzo M., Sciortino M.T., Pennisi R. (2021). In Vitro Anti-Epstein Barr Virus Activity of *Olea europaea* L. Leaf Extracts. Plants.

[31] Bisignano C., Mandalari G., Smeriglio A., Trombetta D., Pizzo M.M., Pennisi R., Sciortino M.T. (2017). Almond Skin Extracts Abrogate HSV-1 Replication by Blocking Virus Binding to the Cell. Viruses.

[32] (2012). Institute Performance Standards for Antimicrobial Susceptibility Testing.

[33] (2008). Clinical and Laboratory Standards Institute Reference Method for Broth Dilution Antifungal Susceptibility Testing of Yeasts, Approved Standard.

[34] Gómez-Caravaca A.M., Verardo V., Toselli M., Segura-Carretero A., Fernández-Gutiérrez A., Caboni M.F. (2013). Determination of the major phenolic compounds in pomegranate juices by HPLC−PDAPDA−ESI-MS. J. Agric. Food Chem..

[35] Li J., He X., Li M., Zhao W., Liu L., Kong X. (2015). Chemical fingerprint and quantitative analysis for quality control of polyphenols extracted from pomegranate peel by HPLC. Food Chem..

[36] Brighenti V., Groothuis S.F., Prencipe F.P., Amir R., Benvenuti S., Pellati F. (2017). Metabolite fingerprinting of *Punica granatum* L. (pomegranate) polyphenols by means of high-performance liquid chromatography with diode array and electrospray ionization-mass spectrometry detection. J. Chromatogr. A.

[37] Russo M., Fanali C., Tripodo G., Dugo P., Muleo R., Dugo L., De Gara L., Mondello L. (2018). Analysis of phenolic compounds in different parts of pomegranate (*Punica granatum*) fruit by HPLC-PDA-ESI/MS and evaluation of their antioxidant activity: Application to different Italian varieties. Anal. Bioanal. Chem..

[38] Ding W., Wang H., Zhou Q., Wu C., Gao X., Cheng X., Tian L., Wang C. (2019). Simultaneous determination of polyphenols and triterpenes in pomegranate peel based on high-performance liquid chromatography fingerprint by solvent extraction and ratio blending method in tandem with wavelength switching. Biomed. Chromatogr..

[39] Hernández-Corroto E., Marina M.L., García M.C. (2019). Extraction and identification by high resolution mass spectrometry of bioactive substances in different extracts obtained from pomegranate peel. J. Chromatogr. A.

[40] Swilam N., Nematallah K.A. (2020). Polyphenols profile of pomegranate leaves and their role in green synthesis of silver nanoparticles. Sci. Rep..

[41] Yu M., Gouvinhas I., Barros A. (2021). Variation of the Polyphenolic Composition and Antioxidant Capacity of Freshly Prepared Pomegranate Leaf Infusions over One-Day Storage. Antioxidants.

[42] Abid M., Yaich H., Cheikhrouhou S., Khemakhem I., Bouaziz M., Attia H., Ayadi M.A. (2017). Antioxidant properties and phenolic profile characterization by LC-MS/MS of selected Tunisian pomegranate peels. J. Food Sci. Technol..

[43] Govindappa M., Tejashree S., Thanuja V., Hemashekhar B., Srinivas C., Nasif O., Raghavendra V.B. (2021). Pomegranate fruit fleshy pericarp mediated silver nanoparticles possessing antimicrobial, antibiofilm formation, antioxidant, biocompatibility and anticancer activity. J. Drug Deliv. Sci. Technol..

[44] Zeghad N., Ahmed E., Belkhiri A., Heyden Y.V., Demeyer K. (2019). Antioxidant activity of Vitis vinifera, *Punica granatum*, Citrus aurantium and Opuntia ficus indica fruits cultivated in Algeria. Heliyon.

[45] Elbatanony M.M., El-Feky A.M., Hemdan B.A., El-Liethy M.A. (2019). Assessment of the antimicrobial activity of the lipoidal and pigment extracts of *Punica granatum* L. leaves. Acta Ecol. Sin..

[46] Sun S., Huang S., Shi Y. (2021). Extraction, isolation, characterization and antimicrobial activities of non-extractable polyphenols from pomegranate peel. Food Chem..

[47] Peršurić Ž., Saftić Martinović L., Malenica M., Gobin I., Pedisić S., Dragović-Uzelac V., Kraljević Pavelić S. (2020). Assessment of the Biological Activity and Phenolic Composition of Ethanol Extracts of Pomegranate (*Punica granatum* L.) Peels. Molecules.

[48] Kupnik K., Primožič M., Vasić K., Knez Ž., Leitgeb M. (2021). A Comprehensive Study of the Antibacterial Activity of Bioactive Juice and Extracts from Pomegranate (*Punica granatum* L.) Peels and Seeds. Plants.

[49] Trabelsi A., El Kaibi M.A., Abbassi A., Horchani A., Chekir-Ghedira L., Ghedira K. (2020). Phytochemical Study and Antibacterial and Antibiotic Modulation Activity of *Punica granatum* (Pomegranate) Leaves. Scientifica.

[50] Ismail T., Sestili P., Akhtar S. (2012). Pomegranate peel and fruit extracts: A review of potential anti-inflammatory and anti-infective effects. J. Ethnopharmacol..

[51] Sharma J., Maity A. (2010). Pomegranate phytochemicals: Nutraceutical and therapeutic values. Fruit Veg. Cereal Sci. Biotech..

[52] Arunkumar J., Rajarajan S. (2018). Study on antiviral activities, drug-likeness and molecular docking of bioactive compounds of *Punica granatum* L. to Herpes simplex virus–2 (HSV-2). Microb. Pathog..

[53] Moradi M.T., Karimi A., Shahrani M., Hashemi L., Ghaffari-Goosheh M.S. (2019). Anti-Influenza Virus Activity and Phenolic Content of Pomegranate (*Punica granatum* L.) Peel Extract and Fractions. Avicenna J. Med. Biotechnol.

[54] Neurath A.R., Strick N., Li Y.Y., Debnath A.K. (2005). *Punica granatum* (Pomegranate) juice provides an HIV-1 entry inhibitor and candidate topical microbicide. Ann. N. Y. Acad. Sci..

[55] Suručić R., Travar M., Petković M., Tubić B., Stojiljković M.P., Grabež M., Šavikin K., Zdunić G., Škrbić R. (2021). Pomegranate peel extract polyphenols attenuate the SARS-CoV-2 S-glycoprotein binding ability to ACE2 Receptor: In silico and in vitro studies. Bioorg. Chem..

[56] Salles T.S., Meneses M.D.F., Caldas L.A., Sá-Guimarães T.E., de Oliveira D.M., Ventura J.A., Azevedo R.C., Kuster R.M., Soares M.R., Ferreira D.F. (2021). Virucidal and antiviral activities of pomegranate (*Punica granatum*) extract against the mosquito-borne Mayaro virus. Parasites Vectors.

[57] Acquadro S., Civra A., Cagliero C., Marengo A., Rittà M., Francese R., Sanna C., Bertea C., Sgorbini B., Lembo D. (2020). *Punica granatum* leaf ethanolic extract and ellagic acid as inhibitors of zika virus infection. Planta Med..

[58] Angamuthu D., Purushothaman I., Kothandan S., Swaminathan R. (2019). Antiviral study on *Punica granatum* L., *Momordica charantia* L., *Andrographis paniculate* Nees, and *Melia azedarach* L., to Human Herpes Virus-3. Eur. J. Integr. Med..

[59] Venusova E., Kolesarova A., Horky P., Slama P. (2021). Physiological and Immune Functions of Punicalagin. Nutrients.

[60] Lin L.T., Chen T.Y., Lin S.C., Chung C.Y., Lin T.C., Wang G.H., Anderson R., Lin C.C., Richardson C.D. (2013). Broad-spectrum antiviral activity of chebulagic acid and punicalagin against viruses that use glycosaminoglycans for entry. BMC Microbiol..

